# Potent induction of trained immunity by *Saccharomyces cerevisiae* β-glucans

**DOI:** 10.3389/fimmu.2024.1323333

**Published:** 2024-02-13

**Authors:** Patricia Vuscan, Brenda Kischkel, Aikaterini Hatzioannou, Efrosyni Markaki, Andrei Sarlea, Maria Tintoré, Jordi Cuñé, Panayotis Verginis, Carlos de Lecea, Triantafyllos Chavakis, Leo A.B. Joosten, Mihai G. Netea

**Affiliations:** ^1^ Department of Internal Medicine and Radboud Institute for Molecular Life Sciences, Radboud University Medical Center, Nijmegen, Netherlands; ^2^ Institute for Clinical Chemistry and Laboratory Medicine, University Hospital and Faculty of Medicine, TU Dresden, Dresden, Germany; ^3^ Laboratory of Immune Regulation and Tolerance, Medical School, University of Crete, Heraklion, Greece; ^4^ R&D Department, AB Biotek Human Nutrition and Health, Barcelona, Spain; ^5^ Department of Medical Genetics, Iuliu Hatieganu University of Medicine and Pharmacy, Cluj-Napoca, Romania; ^6^ Department for Immunology and Metabolism, Life and Medical Sciences Institute (LIMES), University of Bonn, Bonn, Germany

**Keywords:** innate immunity, probiotics, melanoma, bladder cell carcinoma, immunotherapy

## Abstract

*Candida albicans* cell wall component β-glucan has been extensively studied for its ability to induce epigenetic and functional reprogramming of innate immune cells, a process termed *trained immunity*. We show that a high-complexity blend of two individual β-glucans from *Saccharomyces cerevisiae* possesses strong bioactivity, resulting in an enhanced trained innate immune response by human primary monocytes. The training required the Dectin-1/CR3, TLR4, and MMR receptors, as well as the Raf-1, Syk, and PI3K downstream signaling molecules. By activating multiple receptors and downstream signaling pathways, the components of this β-glucan preparation are able to act synergistically, causing a robust secondary response upon an unrelated challenge. In *in-vivo* murine models of melanoma and bladder cell carcinoma, pre-treatment of mice with the β-glucan preparation led to a significant reduction in tumor growth. These insights may aid in the development of future therapies based on β-glucan structures that induce an effective trained immunity response.

## 1 Introduction

One of the most abundant forms of polysaccharides found in nature, β-glucans, are glucose polymers that occur in the cell walls of fungi, yeasts, and bacteria. β-glucans are recognized by pattern recognition receptors on the surface of immune cells, and they have a diverse range of immuno-stimulatory properties (1, 2). Through their immunostimulatory capacity, various preparations of β-glucan have been tested and used effectively in cancer immunotherapy across East Asia, primarily in Japan (3). Both animal models and human studies have suggested an important anti-tumor potential of β-glucans, either directly or by synergizing with other immunotherapeutic approaches such as checkpoint inhibitors (4–6). It is therefore important to have a complete understanding of the specific immunologic mechanisms underlying yeast-derived β-glucans, in order to make efficient use of their immuno-stimulatory potential.

*Candida albicans* cell wall component β-glucan is a pathogen-associated molecular pattern (PAMP) with potent immunomodulatory effects on innate immunity (1). Specifically, it has been demonstrated that β-glucans induce trained innate immunity (TII): human monocytes pre-exposed to β-glucan exhibit an enhanced immune response upon secondary stimulation with various secondary stimuli (7, 8). This effect is mediated by the C-type lectin receptor Dectin-1 (9) and complement receptor 3 (CR3) (7). Subsequent activation of the Akt/mTOR/Hif1α pathway triggers epigenetic rewiring in monocytes/macrophages, causing the upregulation of genes that code for pro-inflammatory cytokines, such as tumor necrosis factor (TNF) and interleukin-1β (IL-1β) and IL-6 (10–12). Moreover, TII induced by β-glucans is also accompanied by cellular metabolism reprogramming, including a shift from oxidative phosphorylation to aerobic glycolysis, called the Warburg effect (13, 14). In addition, a recent study demonstrated that TII induced by β-glucans in mice also involves the reprogramming of hematopoietic progenitor cells in the bone marrow, leading to increased production of myeloid cells with an enhanced capacity to respond to stimuli in the periphery (15, 16).

β-glucans are complex molecules, with significant variability in conformational complexity, branching type and degree, all structural properties that influence their biological activity (7, 17). The most studied immuno-active structure of β-glucan has a backbone structure consisting of linear β-1,3-linked D-glucose molecules (β-1,3-(D)-glucan). Branches of varying sizes can occur at various intervals along the backbone, the two main groups being the 1→4 or 1→6 glycosidic chains (18). It has been previously suggested that a higher degree of structural complexity is associated with the more potent immunomodulatory and anti-cancer effects of glucans (19).

An in-depth analysis of various β-glucans in terms of their capacity to induce trained immunity has not been performed till now. In the present study, six different fractions of β-glucan from *Saccharomyces cerevisiae*, together with a blend of probiotic strains, were evaluated for their potential to induce trained immunity. We show that preparation ABB i16 has superior bioactivity in inducing TII in human monocytes compared to β-glucan isolated from *C. albicans*, other yeast β-glucan fractions, or the probiotic strains. In addition, PRRs and metabolic enzymes that play a role in the induction of TII by fraction ABB i16 are identified.

## 2 Methods

### 2.1 β-glucan preparations

β-1,3-(D)-Glucan from *C. albicans* (*Ca* β-glucan) was kindly provided by Professor David Williams (College of Medicine, Johnson City, USA). We used 6 different preparations of β-1,3/1,6-glucan extracted from *S. cerevisiae* (*Sc* β-glucan): 4 preparations- namely ABB i24, ABB i25, and ABB i29, and a combination of ABB i24 and ABB i25 named ABB i16 were provided by AB Biotek HNH, (Barcelona, Spain). CC1 (M-Gard particulate) and CC2 (Wellmune) were purchased as commercially available β-glucans from *S. cerevisiae*. A full description of the preparations can be found in **Table 1**.

**Table 1:** Characteristics of the various β-glucan preparations used in this study.

Code	Origin	Description	Chemical structure	Glucan (% d.m.)	Mannan (% d.m.)	Protein (% d.m.)	Fat (% d.m.)
***Ca* β-glucan**	*C. albicans*		-1,3-(D)-Glucan				
**ABB i24**	*S. cerevisiae*		-1,3/1,6- Glucan	>50.00	≤6.00	≤8.00	18.0
**ABB i25**	*S. cerevisiae*		-1,3/1,6- Glucan	>70.00	≤4.00	≤5.00	10.0
**ABB i16**	*S. cerevisiae*	Blend of ABB i24 + ABB i25	-1,3/1,6- Glucan	≥68.00	≤4.50	≤5.50	17.0
**ABB i29**	*S. cerevisiae*		-1,3/1,6- Glucan	≥45.00	<1.24	<16.00	8.5
**CC1** **(M-Gard^®^)**	*S. cerevisiae*	Commercial control	-1,3/1,6- Glucan	>75.00	<1.20*	≤3.50	4.0
**CC2 (Wellmune^®^)** (20)	*S. cerevisiae*	Commercial control	-1,3/1,6- Glucan	81.00	0.5%	<3.50	<10.0

d.m.: dry matter. n.a. not available. *this value was measured % OS.

### 2.2 Probiotic strains

The blend of probiotic strains was obtained from commercial sources. The strains included in the product were *Lactobacillus plantarum* CECT 30292, *L. plantarum* CECT 7484, *L. plantarum* CECT 7485, and *L. plantarum* CECT 7483.

### 2.3 Isolation of human primary monocytes

Peripheral blood mononuclear cells (PBMCs) were isolated from buffy coats after informed consent was obtained from healthy adult donors, from Sanquin Bloodbank (Nijmegen, The Netherlands). Isolation of PBMCs was performed by dilution of blood in sterile phosphate-buffered saline (PBS) and density centrifugation over Ficoll-Paque, (GE Healthcare, Zeist, The Netherlands). PBMCs were then washed twice with cold PBS and resuspended in RPMI 1640 culture medium Dutch Modification (Gibco, Life Technologies, MA) supplemented with 10 *μ*g/mL gentamicin (Centafarm, Etten-Leur, the Netherlands), 10 mM GlutaMAX, and 10 mM pyruvate (Life Technologies). Percoll isolation of monocytes was performed as described previously (21). Cells were resuspended in culture medium and counted using the Sysmex haematology analyser (XN-450). The purity of the isolated monocytes was approximately 50-70%, allowing the persistence of low percentages of lymphocytes and NK-cells to be present. This method confers an *in-vivo* situation in our system, more closely resembling the real conditions found in the human body compared to highly purified cell populations.

### 2.4 *In-vitro* induction of trained immunity in monocytes

The PBMCs were resuspended in culture medium and the concentration was adjusted to 5 × 10^6^ cells/mL (or 500,000 cells in 100 μL/well) and added to flat-bottom 96-well plates. After incubation for 1h at 37°C, non-adherent cells were washed away with warm PBS, and the remaining attached monocytes were stimulated with *Ca* β-glucan, with 10, 5, 2, or 1 μg/mL of various *Sc* β-glucan fractions, as well as 1 μg/mL of the probiotic strains blend for 24h. To eliminate the possibility of lipopolysaccharide (LPS) contamination, all the fractions of β-glucans were pre-incubated for 1h with and without 2 μg/mL of polymyxin B (Bedford Laboratories, Bedford, OH). After 24h, monocytes were washed with warm PBS to remove the stimulus and allowed to rest in medium for 5 days. Subsequently, the cells were restimulated with *Escherichia coli* LPS (10 ng/mL, serotype 055:B5, Sigma-Aldrich) for 24h.

### 2.5 Receptor and intracellular signaling inhibition

For receptor and intracellular signaling pathways inhibition experiments, 5 × 10^6^ monocytes/mL (or 5 × 10^5^ cells in 100 μL/well) were added to flat-bottom 96-well cell culture plates and pre-incubated for 1h with antibodies and small molecule inhibitors. PRRs were blocked using 10 μg/mL of anti-Dectin-1, 10 μM of anti-CR3 antibodies, and 10 μg/mL mannose receptor (MR)-blocking antibodies along with their respective isotype control antibodies, 10 μg/mL of Mouse IgG and Goat IgG (R&D Systems), and 20 ng/mL *Bartonella quintana* LPS (*Bart.* LPS, TLR4 antagonist). For the inhibition of downstream signaling mediators, monocytes were pre-incubated with 50 nM of spleen tyrosine kinase (Syk) inhibitor (574711, Sigma), 1 µM of noncanonical serine-threonine kinase Raf-1 inhibitor (GW5704, Sigma), and 10 µM of phosphoinositide-3 kinase (PI3K) inhibitor Wortmannin (SL-2052, R&D Systems). Cells were then stimulated at 37°C, 5% CO_2_ with 2 μg/mL -1,3-(D)-Glucan (Ca b-glucan) and 2 μg/mL of ABB i16. The culture contained the inhibitors both during blocking (1h) and stimulation periods (24h). Supernatants were collected in the first 24 hours of stimulation to measure the production of pro-inflammatory cytokines. Next, cells were washed with warm PBS, then allowed to rest in medium (containing 10% human pooled serum) for 5 days. On the 6^th^ day, the cells were restimulated with *Escherichia coli* LPS (10 ng/mL, serotype 055:B5, Sigma-Aldrich) for 24h, and supernatants were collected for cytokine production measurements.

### 2.6 Cytokine measurements

Cytokine production measurements were performed using commercial ELISA kits according to the manufacturer’s protocol (R&D Systems, Minneapolis, USA). TNF and IL-6 production was measured from supernatants collected after 24h or 7 days of stimulation.

### 2.7 Mouse experiments

Male C57BL/6J mice were purchased from Jackson Laboratory. The mice were housed under specific pathogen-free conditions on a standard 12/12 h light/dark cycle and were used at the age of 8-10 weeks. Food and water were provided *ad libitum*. Solid tumor induction was performed as described previously (22, 23). Briefly, injection of mice was done subcutaneously (s.c.) into the right flank, using 3 x 10^5^ B16-F10 melanoma cancer cells. In other experiments, 7.5 x 10^5^ MB49 bladder cancer cells were subcutaneously injected. To investigate the impact of the induction of trained innate immunity on tumor development, mice underwent pre-treatment intraperitoneally (i.p.) with a single dose of 1 mg of the ABB i16 β-glucan preparation or with vehicle alone used as control, and tumor cell injection was performed 7 days later. The volume of the palpable tumors was monitored and weighing of the tumors and flow cytometry analysis were performed at the end of the experiment (14 days following tumor inoculation in the case of B16F10 melanoma or 16 days following tumor inoculation in the case of MB49 bladder cancer cells). Mice were excluded if pre-established exclusion criteria were fulfilled, for example, appearance of ulcerated tumors as an animal protocol-defined endpoint or, in the cases that no tumor growth was observed (23–26). Animal experiments were approved either by the Landesdirektion Sachsen, Dresden Germany, or the Institutional Committee of Protocol Evaluation of the IMBB together with the Directorates of Agricultural Economy and Veterinary, Region of Crete, Greece.

### 2.8 Flow cytometry of mouse immune cells

Cell analysis was conducted using a BD LSRFortessa (BD, Heidelberg, Germany). Single-cell suspensions from tumors were prepared by incubating dissected tissue for 45 minutes at 37°C in RPMI culture medium supplemented with 0.25 mg/mL DNase I (Sigma-Aldrich) and 1 mg/mL collagenase D (Roche). For mouse spleens and lymph nodes, homogenization was performed, and splenic single-cell suspensions were obtained following erythrocyte lysis using red blood cell lysis buffer (eBioscience). For cell surface phenotype analysis, anti-CD11b-BV510 (clone M1/70), anti-CD45-PercP/Cy5.5 (clone 30-F11), anti-CD3-APC (clone 17A2), anti-CD4-PE (clone RM4-4), anti-CD8a-PECy7 (clone 53-6.7), anti-Ly6G-APC (clone 1A8), anti-Ly6C-BV421 (clone HK1.4), anti-CD11c-PE (clone N418), anti-NK1.1-FITC (clone PK136) were used. For exclusion of dead cells, 7AAD or LIVE/DEAD™ Fixable Aqua was applied. Data analysis was performed using FlowJo (Tree Star) software.

### 2.9 Statistical analysis

Differences between experimental conditions were analysed using Mann-Whitney U test or Wilcoxon matched-pair signed-rank test, as indicated. We used three different human blood samples per experiment and we performed 2 independent experiments. For experiments with a sample size <4, no statistical analysis was performed due to the small sample size. Statistical testing was performed using the GraphPad Prism software (San Diego, CA, USA). Data are given as mean standard error of the mean (SEM), and values of *p < 0.05, **p < 0.01, ***p < 0.001, or ****p < 0.0001 were considered statistically significant.

## 3 Results

### 3.1 Trained immunity induction by different fractions of β-glucans in human primary monocytes

We used β-glucans with different molecular structures (**Table 1**), derived from the cell walls of *Saccharomyces cerevisiae* and *Candida albicans*, to investigate cytokine responses of human primary monocytes and macrophages. The pre-incubation of monocytes with or without polymyxin B did not affect the production of TNF and IL-6, demonstrating that all stimuli were free of residual lipopolysaccharide (LPS). Used as a positive control, LPS-induced TNF secretion was decreased significantly in the presence of polymyxin B (**Supplementary Figure 1**).

**Figure 1:**
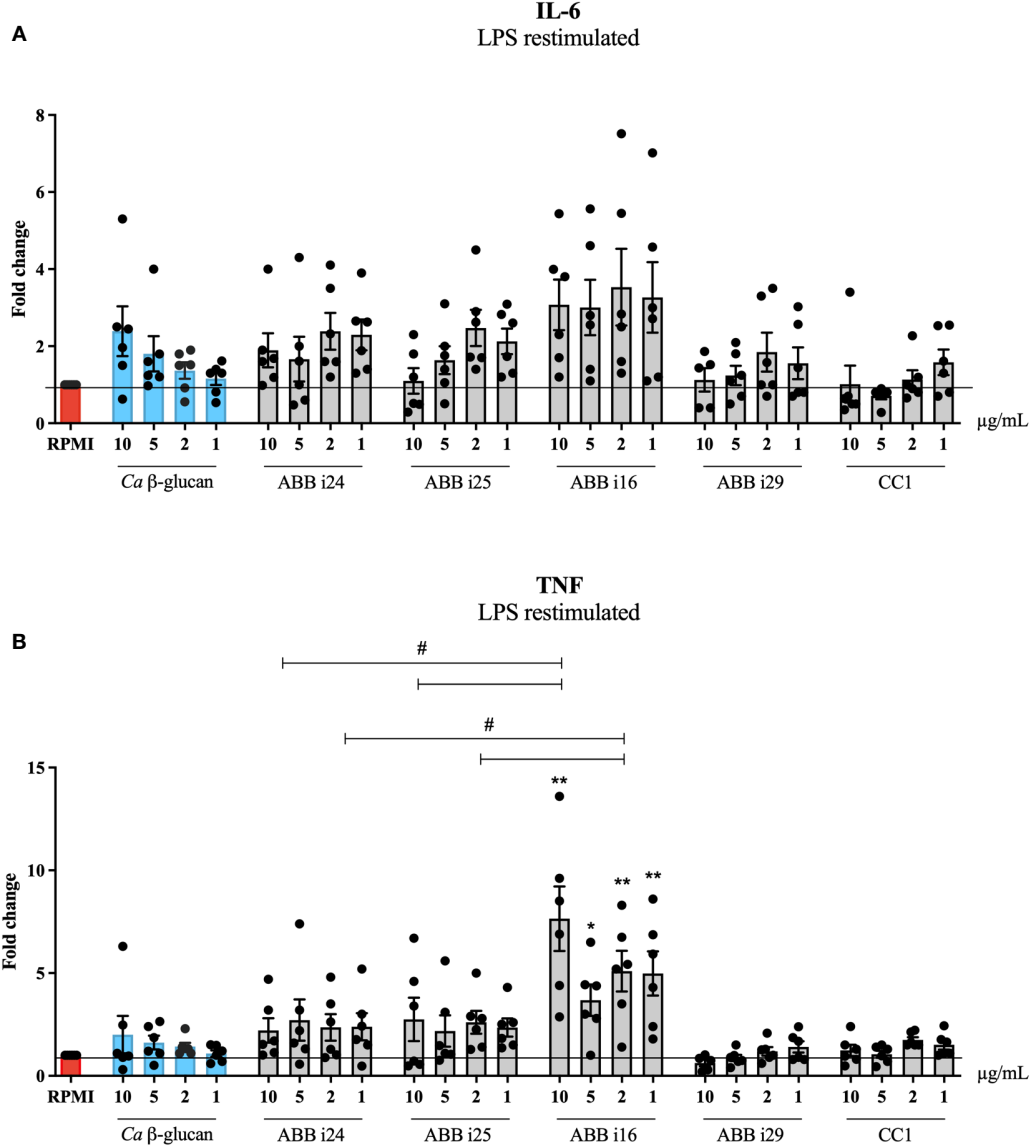
Trained immunity induction by 5 different fractions of β-glucans from *S. cerevisiae*. Monocytes were trained with 1, 2, 5, and 10µg/mL of *Ca* β-glucan or *Sc* β-glucans for 24 hours. On day 6 after resting, macrophages were re-stimulated with 10 ng/mL of LPS and incubated for 24 hours. IL-6 **(A)** and TNF **(B)** production was measured in supernatants by ELISA, n=6. Data in dot plots in bar diagrams are represented as mean ± SEM. Statistical analysis was performed by Mann-Whitney U test. *p < 0.05, **p < 0.01; comparing with *Ca* β-glucan as control, or ^#^p 0.05 differs between ABB i24, ABB i25 and ABB i16-stimulated conditions.

We exposed human primary monocytes to different concentrations (10, 5, 2, and 1 µg/mL) of six different preparations of *S. cerevisiae* β-glucans, namely ABB i24, ABB i25, ABB i16, ABB i29, and the CC1 and CC2 commercial controls. As a positive control, we used *C. albicans* β-glucan, since its abilities to induce trained immunity have already been well-established (7), and the negative control consisted of RPMI medium condition. The exposure of monocytes to both *C. albicans* and *S. cerevisiae* β-glucans led to an increase in the production of TNF and IL-6 upon LPS restimulation on day 6, measured as the fold increase normalized to RPMI (**Figures 1A, B**). For *C. albicans* β-glucan, the fold increase of TNF and IL-6 was dose-dependent, with the highest concentration of 10 µg/mL inducing the highest increase. As shown in **Figure 1B**, the concentrations of 10, 2, and 1 µg/mL of ABB i16 induced a 2-times higher fold increase in TNF production, compared to the respective concentrations of *Ca* β-glucan, and a one-time higher fold increase compared to the corresponding ABB i24 and ABB i25. Here, the lowest overall fold increase in cytokine production was observed for CC1-stimulated cells.

**Figure 2:**
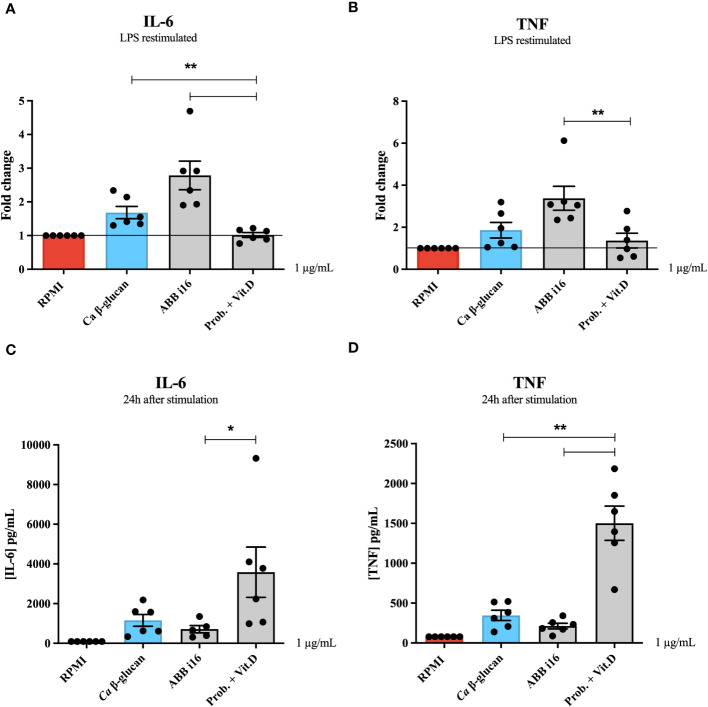
24h cytokine production and trained immunity induction by ABB i16, Ca β-glucan, and the probiotic blend. Human monocytes were trained with *Ca* β-glucan, *Sc* β-glucans, or the probiotic blend for 24 hours. On day 6 after resting, macrophages were re-stimulated with 10 ng/mL of LPS and incubated for 24 hours. IL-6 **(A)** and TNF **(B)** production was measured in supernatants by ELISA, n=6. Cells were exposed to *Ca* β-glucan, *Sc* β-glucans, or the probiotic blend. In the first 24 hours, IL-6 **(C)** and TNF **(D)** production was also measured in supernatants by ELISA, n=6. Data in dot plots in bar diagrams are represented as mean ± SEM. Statistical analysis was performed by Mann-Whitney U test. *p < 0.05, **p < 0.01; differs between probiotic preparation, ABB i16, and ABB i29-stimulated conditions.

Similarly, we also assessed how ABB i16, which exhibited the strongest overall cytokine production, is able to induce trained immunity compared to a different stimulus, namely a blend of probiotic strains. (**Figures 2A, B**). Human monocytes exposed to 1 µg/mL of the ABB i16 preparation showed a 2-times higher fold increase in both IL-6 and TNF production, in comparison with the corresponding concentration of the probiotics blend (Prob. + Vit.D) upon LPS re-challenge (**Figures 2A, B**). Similarly, the CC2 Wellmune β-glucan was only poorly able to induce trained immunity (**Supplemetary Figure 2**).

### 3.2 Differential production of TNF and IL-6 after 24h stimulation

**Figure 3:**
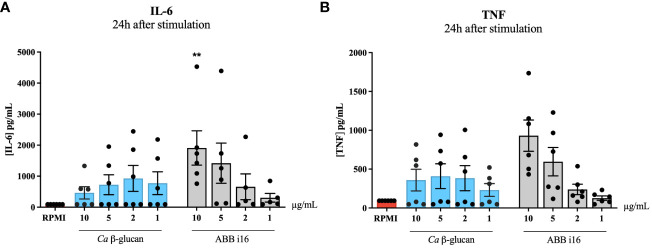
24h stimulation cytokine production. Monocytes were primed with 1, 2, 5 and 10 µg/mL of *Ca* β-glucan or *Sc* β-glucans for 24 hours. After 24h stimulation, supernatants were collected and **(A)** IL-6 and **(B)** TNF production was measured in supernatants by ELISA, n=6. Data in dot plots in bar diagrams are represented as mean ± SEM. Statistical analysis was performed by Mann-Whitney U test. *p 0.05, **p < 0.01 compared to *Ca* β-glucan control.

The previous experiment determined that the strongest inducer of trained immunity is ABB i16 (**Figure 1**). To assess whether induction of trained immunity is associated with the capacity of β-glucans to induce strong inflammation, we first measured the cytokine production after 24h of stimulation with 10, 5, 2, or 1 µg/mL of ABB i16 and *Ca* β-glucan as the control stimulus (**Figures 3A, B**). In this context, we observed that monocytes exposed to 10 µg/mL of ABB i16 induced higher concentrations of IL-6 compared to the corresponding concentrations of *Ca* β-glucan (**Figure 3A**). However, when using the lower concentrations, the difference in the production of TNF and IL-6 by the two stimuli was not statistically significant (**Figures 3A, B**). In a subsequent experiment, our data showed that stimulation with the concentration of 1 µg/mL of probiotics was able to induce significantly higher levels of TNF and IL-6 (**Figures 2C, D**). This evidence suggests that induction of trained immunity is dependent of the capacity of β-glucan or probiotics preparations to induce a balanced inflammatory response in the first 24 hours.

### 3.3 Pattern recognition receptors (PRRs) and downstream signaling molecules involved in triggering the immune response by *Sc* β-glucan ABB i16

**Figure 4:**
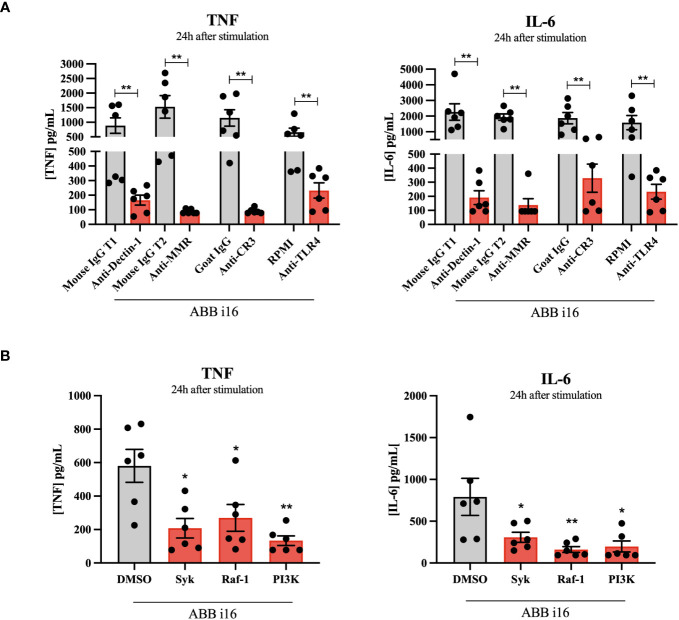
Identification of *Sc* β-glucan receptor pathways and intracellular signaling pathways. Monocytes were pre-incubated for 1h with RPMI, or isotype antibodies IgG Mouse and IgG2b Goat (positive controls), or anti-hDectin-1, anti-MMR, anti-CR3 blocking antibodies and TLR-4 inhibitor *Bart*.-LPS **(A)**, or DMSO (positive control), Syk kinase inhibitor, Raf-1 inhibitor GW5074, and PI 3-kinase inhibitor SL-2052 **(B)**, then stimulated for 24 hours with ABB i16. TNF and IL-6 production was measured in supernatants by ELISA, n=6. The Mouse IgG T1 and T2 conditions represent two different trials comparing neutralizing antibodies with their corresponding isotype control, with different sets of donors. Data in dot plots in bar diagrams are represented as mean ± SEM. Statistical analysis was performed by Mann-Whitney U test. *p < 0.05, **p < 0.01 compared to control.

After ABB i16 was determined to have the strongest stimulatory effect on trained immunity induction (**Figure 1**), we chose the concentration of 2 µg/mL for subsequent experiments in which we investigated which receptors are involved in the induction of both inflammation and trained immunity. First, monocytes were incubated in the presence of different receptor and pathway-blocking antibodies and inhibitors (and appropriate positive controls) one hour prior to the addition of ABB i16. The inhibition of Dectin-1, MMR, CR3, and TLR4 blocked the uptake of ABB i16, leading to a significant decrease in both TNF and IL-6 production in the first 24 hours after ABB i16 stimulation, in comparison to their corresponding controls (**Figure 4A**). Following exposure of monocytes to Syk and Raf-1 inhibitors, we observed a decrease in the production of both TNF and IL-6, with a stronger inhibition of IL-6, after stimulation with ABB i16 compared to the control condition (**Figure 4B**). Furthermore, β-glucan recognition in innate immune cells by Dectin-1 and subsequent intracellular signalling is followed by stimulation of downstream pathways, such as phosphoinositide-3 kinase (PI3K) (27). The blockade of this downstream cellular metabolism mediator by a PI3K-specific inhibitor significantly decreased pro-inflammatory cytokines TNF and IL-6 production induced by ABB i16 after 24 hours of stimulation (**Figure 4B**).

**Figure 5:**
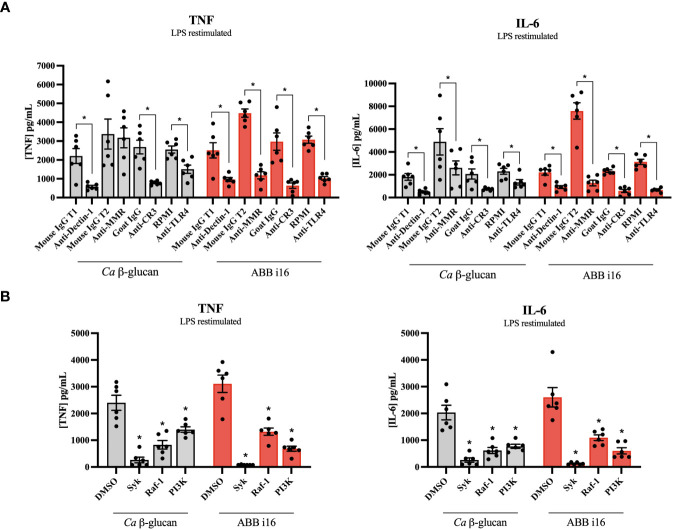
Receptors and signaling pathways involved in ABB i16 and Ca β-glucan trained immunity induction. 1h with RPMI, or isotype antibodies IgG Mouse and IgG2b Goat (positive controls), or anti-hDectin-1, anti-MMR, anti-CR3 blocking antibodies and TLR-4 inhibitor *Bart*.-LPS **(A)**, or DMSO (positive control), Syk kinase inhibitor, Raf-1 inhibitor GW5074, and PI 3-kinase inhibitor SL-2052 **(B)**, then primed for 24 hr with *Ca* β-glucan or ABB i16. Subsequently washed, rested for 5 days, and at day 6 restimulated for 24h with 10 ng/mL LPS. TNF and IL-6 production was measured in supernatants by ELISA, n=6. The Mouse IgG T1 and T2 conditions represent two different trials comparing neutralizing antibodies with their corresponding isotype control, with different sets of donors. Data in dot plots in bar diagrams are represented as mean ± SEM. Statistical analysis was performed by Wilcoxon matched-pair signed-rank test. *p < 0.05 compared to control.

Second, we sought to investigate if these receptors and their downstream signaling molecules could influence the TII response. The TII phenotype was assessed by the ability of trained monocytes to release TNF and IL-6 after LPS re-challenge on day 6. The blockade of Dectin-1, CR3, MMR, and TLR4 during the first 24h of training led to a significant decrease in the cytokine production by cells primed with ABB i16 of both TNF and IL-6 on day 6 after LPS restimulation, in comparison with their respective isotype control conditions (**Figure 5A**). On the other hand, blocking of the MMR did not interfere with TNF production upon *Ca* β-glucan stimulation. The exposure of monocytes to the Syk inhibitor during the trained immunity induction phase of the experiment led to a significant decrease in the later induction of TNF and IL-6 in ABB i16-trained macrophages compared to the control condition (**Figure 5B**). A similar, though less pronounced, downregulation of TNF and IL-6 production was observed after blocking the Dectin-1 signaling pathway component Raf-1 (**Figure 5B**). Subsequently, we assessed whether the inhibition of the PI3K signaling cascade also plays a role in TII induction by ABB i16. Indeed, after stimulation of monocytes pre-incubated with a PI3K inhibitor, the production of TNF and IL-6 pro-inflammatory cytokines was notably lower (**Figure 5B**).

### 3.4 Effect of trained innate immunity induction in *in-vivo* murine tumor models

**Figure 6:**
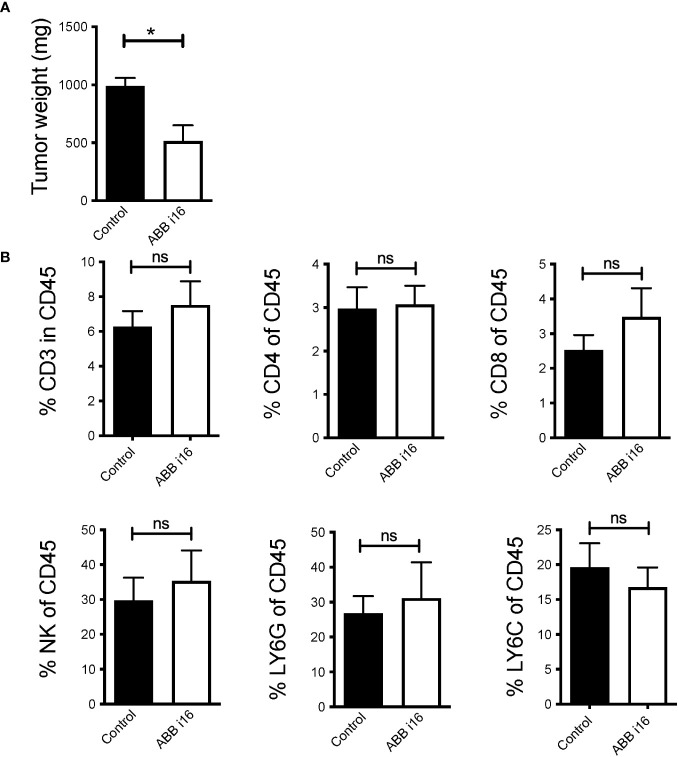
ABB i16 induced-trained innate immunity prevents tumor growth in mice. Mice were i.p. injected with 1mg of ABB i16 beta-glucan or received a control injection 7 days before s.c. inoculation of B16F10 cells. 14 days thereafter tumors were extracted and analyzed. **(A)** The tumor weight at day 14 is shown. **(B)** The percentages of tumor infiltrating CD3^+^ T cells (live CD45^+^CD3^+^NK1.1^-^), CD4^+^ T cells (live CD45^+^CD3^+^NK1.1^-^CD4^+^), CD8^+^ T cells (live CD45^+^CD3^+^NK1.1^-^CD8^+^), natural killer cells (NK; live CD45^+^CD3^-^NK1.1^+^), LY6G^+^ neutrophils (live CD45^+^CD11c^-^CD11b^+^Ly6G^+^Ly6C^-^) and LY6C^+^ monocytes/macrophages (live CD45^+^CD11c^-^CD11b^+^Ly6G^-^Ly6C^+^) in the total population of CD45^+^ infiltrating cells, analyzed 14 days after B16F10 inoculation, are shown. Data are mean ± SEM; n=5-7; * p < 0.05.

It was previously established that treatment of mice with β-glucan prior to tumor inoculation leads to significantly suppressed tumor growth (23). In order to study whether TII induced by ABB i16 β-glucan administration can also prevent tumor growth, mice were injected with a single dose of ABB i16. After 7 days, the mice were subcutaneously administered either with B16-F10 melanoma cells or with MB49 bladder cancer cells. ABB i16-induced TII, as compared to control treatment, significantly reduced melanoma (**Figure 6A**) and bladder cancer growth (**Supplementary Figure 3**). β-glucan did not alter the percentages of tumor infiltrating CD3^+^ T lymphocytes (live CD45^+^CD3^+^NK1.1^-^), CD4^+^ T cells (live CD45^+^CD3^+^NK1.1^-^CD4^+^), cytotoxic CD8^+^ T cells (live CD45^+^CD3^+^NK1.1^-^CD8^+^), natural killer cells (live CD45^+^CD3^-^NK1.1^+^), neutrophils (live CD45^+^CD11c^-^CD11b^+^Ly6G^+^Ly6C^-^), or monocytes/macrophages (live CD45^+^CD11c^-^CD11b^+^Ly6G^-^Ly6C^+^) (**Figure 6B**).

## 4 Discussion

In this study, we identify a highly effective new preparation of β-glucan derived from *S. cerevisiae* that induces strong TII in primary human monocytes, resulting in a robust innate response after a secondary challenge with LPS. Furthermore, we identified the receptors and intracellular signalling pathways required to induce TII by ABB i16 and how the components found in its preparation can synergistically influence the trained immune response. Finally, we showed that this β-glucan preparation significantly reduced the growth of tumors in murine models. These findings improve our understanding of the mechanistic aspects through which *S. cerevisiae* β-glucans induce long-term innate immune memory and protective heterologous effects. This information has the potential to contribute to the development of novel pharmacological assets and immunotherapies for the treatment of infections and cancer.

The ability of *C. albicans*-derived β-glucan to induce trained immunity, leading to upregulation of the immune response and long-term protection against non-specific rechallenging, has been described earlier (7). In our study, we used six different *S. cerevisiae*-isolated glucan preparations, as well as a blend of probiotic strains, and compared their abilities to induce TII in relation to their specific compositions. Importantly, our data show that ABB i16 stimulation is able to strongly amplify the production of the pro-inflammatory cytokines TNF and IL-6, therefore potently inducing trained immunity. When assessing this preparation, it can be observed that ABB i16 is a mixture between the individual compounds ABB i24 and ABB i25, obtained using different extraction processes. The observation that ABB i16 is able to induce a significantly higher response compared to each of the individual compounds is likely caused by the synergism between ABB i24 and ABB i25. This hypothesis is supported by our findings that show multiple receptors involved in the trained immunity induction of ABB i16, arguing for multiple pathways being induced, likely by different polysaccharide products. While β-glucans are the major component of the preparations studied, we cannot exclude that other molecules in its composition, such as mannans, may also play a role in the induction of the trained immunity response.

When compared to *Ca* β-glucan, we have found a significantly higher release of TNF and IL-6 by monocytes trained with ABB i16 after subsequent LPS rechallenge. Similarly, ABB i16 induced much stronger trained immunity effects compared with commercially available β-glucan preparations CC1 and CC2. From this, we can hypothesize that ABB i16 *S. cerevisiae* β-glucan-trained cells might possess even stronger protective effects against different pathogens and better activate host defenses. In contrast, the probiotic strain preparations exhibited the weakest ability of trained immunity induction. To gain a better understanding of their mode of action in the induction of trained immunity, we compared the cytokine production in supernatants after initial stimulation with the probiotic blend and ABB i16. Interestingly, an opposite effect has been observed, as the insult with the probiotic strains produced the highest levels of pro-inflammatory cytokines. This inverse effect can be explained by the induction of innate immune ‘tolerance’ by the probiotic preparation. This process is characterized by the inability of a myeloid cell to activate gene transcription mechanisms leading to an upregulated immune response following restimulation (28). It is therefore likely that the initial exposure of the monocytes to the probiotics led to strong initial stimulation, which epigenetically induces tolerance and prevents the later expression of inflammatory genes (29).

As previous studies have demonstrated, *Ca* β-glucan recognition by human innate immune cells is dependent on the Dectin-1 and CR3 receptors (7, 30, 31). Here, we compared the established mechanisms of receptor recognition and intracellular signaling of *Ca* β-glucan with the new preparation of ABB i16. Our findings suggest that the Dectin-1 receptor and its downstream signalling molecules Syk and Raf-1 are required for the production of IL-6 and TNF induced by initial stimulation with ABB i16. Although this fraction may exhibit some differences in molecular structure compared to *Ca* β-glucan, these differences do not seem to influence recognition by PRRs, since both β-glucans require the same pathways for TII induction. Previous research showcased how signalling via the Dectin-1 receptor mediates induction of trained immunity through mTOR pathway activation, an effect mediated by changes in cellular metabolism resulting in the upregulation of glycolysis (32). The cholesterol synthesis pathway has also been described to be essential for the induction of trained immunity after subsequent β-glucan binding to Dectin-1 (14).

The recognition of β-glucan in innate immune cells by Dectin-1 and subsequent intracellular signalling can additionally be mediated by the activation of the PI3K signaling cascade (27). Our data suggest that the PI3K downstream mediator is also activated following the recognition of ABB i16. This reflects the complexity of Dectin-1 signaling that plays a role in the induction of TII by this specific β-glucan preparation. Furthermore, we identified a role for CR3 for the recognition of ABB i16 and subsequent activation of trained immunity.

We also demonstrate that the induction of trained immunity by ABB i16 is dependent on TLR-4, unlike *Ca* β-glucan-trained cells. It has been suggested that Dectin-1 signaling through the Syk pathway synergizes with various TLRs, including TLR2 and TLR4 (33): the dual induction of dectin-1 and TLR4 by ABB i16 may thus explain its potent bioactivity. As also mentioned previously, one possibility is that ABB i16-mediated trained immunity is induced by the recognition of various other components, such as mannans, found in its composition. *S. cerevisiae*-derived mannan activation of pro-inflammatory cytokine production has previously been demonstrated to occur in a TLR4-dependent manner (34). Data from the literature has shown that, in addition to the specific β-glucan receptors, recognition of *C. albicans* O-linked chains by TLR4 induced cytokine secretion in monocytes, which further corroborates our findings (9). Our experiments using neutralizing antibodies further demonstrated an essential role for MMR in the induction of cytokines by ABB i16. In line with previous research, the blocking of MMR led to only partial loss of *Ca* β-glucan-induced cytokine production (35). Consistent with our hypothesis, these findings illustrate how the recognition of mannans by immune cells is driving the heightened TII response upon exposure to ABB i16. In addition, it is known that a higher degree of structural complexity in glucans contributes to increased cytokine production, and therefore a more potent immunostimulatory potential (17). The higher tridimensional structure complexity of the ABB i16 preparation may also contribute to the cooperative activation of multiple receptors, therefore explaining its strong pro-inflammatory potential.

Adding to this, our group and others have previously demonstrated how the TII response can be amplified due to interferon-γ (IFN-γ) production (36–38). Therefore, the persistence of low percentages of lymphocytes and NK-cells in our *in-vitro* model as sources of IFN-γ, alongside monocytes, mirrors the real conditions within the human body more accurately and provides an additional explanation for the heightened trained immunity that was observed in ABB i16 *S. cerevisiae* β-glucan-trained cells.

Most cancer immunotherapies focus on targeting the adaptive branch of immunity, however, recent studies have shown that innate immune cells are also able to exhibit anti-tumor abilities (16, 23). Moreover, these studies have described the molecular mechanisms through which innate immune training by β-glucan can exert powerful anti-tumor effects (23). Our findings demonstrate that the ABB i16 possesses a similar ability; specifically the capacity to decrease tumor growth that was observed following pre-treatment of mice with ABB i16. The fact that the experiments were confirmed in two independent laboratories, in repeated experiments in two models of melanoma and bladder cancer accounts for the reproducibility of the data presented in this study.

Limited studies have looked into the temporal aspects of the trained immunity phenotype, particularly in the context of β-glucan-induced training. Garcia-Valtanen et al. conducted a noteworthy investigation, showcasing that the training effect induced by β-glucan obtained from *C. albicans* began to decline after three weeks *in vivo*, with no detectable effect at 20 days (39). The unique origin of ABB i16, derived from *S. cerevisiae*, coupled with the observed robust antitumor response, suggests the possibility of an extended duration of trained immunity, lasting at least three weeks. However, our study acknowledges the current lack of conclusive evidence regarding the long-term duration of the immune response induced by ABB i16 *in vivo*. To address this limitation, additional experiments are required to confirm the sustained impact of ABB i16-induced training over an extended period. In addition, another limitation of our study concerns the duration of clearance of the ABB i16 β-glucan preparation in the animals, which cannot be determined due to the lack of tools that would allow us to study this.

Although treatment with ABB i16 β-glucan leads to an enhanced immune response and reduced tumor growth in mice, no evidence for changes in the recruitment of total lymphocytes, including CD4+ or CD8+ tumor-infiltrating lymphocytes (TILs), NK cells, and cells of monocytic and granulocytic origin within the CD45+ immune compartment was noted. To address these limitations and enhance the depth of our understanding of the immune mechanisms that ABB i16 possesses, additional experiments focusing on the tumor microenvironment need to be performed. Specifically, the phenotypic changes in tumor-infiltrating myeloid cells, with an emphasis on monocytes and macrophages need to be explored. This analysis should include an examination of cell surface receptors and their activation/polarization states. Such investigations are essential for determining the impact of ABB i16-induced training on immune cell dynamics within the tumor microenvironment.

Furthermore, to gain a more thorough understanding of the mechanisms underlying ABB i16’s anti-tumor effects, restimulation experiments on ex vivo cultured tumor-associated macrophages (TAMs) need to be performed. This should involve assessing their ability to secrete cytokines in both an antigen-specific and non-specific manner. These experiments are anticipated to contribute to a more comprehensive understanding of the observed enhanced anti-tumor responses in trained animals.

In conclusion, our results demonstrate the ability of a newly characterized combination of two β-glucans from *S. cerevisiae* to induce trained immunity in human primary monocytes, resulting in a potent innate host response after a secondary non-specific challenge. Furthermore, we describe how components in this composition engage Dectin-1/CR3, TLR4, and MMR receptors, and collectively activate different signalling pathways. These factors, together with its complex molecular structure, ultimately play a role in the upregulation of the immune response by this *S. cerevisiae* β-glucan fraction. Such potent TII induction potential could contribute to the development of novel treatment strategies for patients with diseases characterized by an aberrant immune response, such as cancer and severe infections.

## Data availability statement

The original contributions presented in the study are included in the article/**Supplementary Material**, further inquiries can be directed to the corresponding author/s.

## Ethics statement

The studies involving humans were approved by CMO Arnhem-Nijmegen (NL32 357.091.10). The studies were conducted in accordance with the local legislation and institutional requirements. The human samples used in this study were acquired from blood obtained from Sanquin Bloodbank (Nijmegen, The Netherlands). Written informed consent for participation was not required from the participants or the participants’ legal guardians/next of kin in accordance with the national legislation and institutional requirements. The animal study was approved by Landesdirektion Sachsen, Dresden Germany, or the Institutional Committee of Protocol Evaluation of the IMBB together with the Directorates of Agricultural Economy and Veterinary, Region of Crete, Greece. The study was conducted in accordance with the local legislation and institutional requirements. No potentially identifiable images or data are presented in this study.

## Author contributions

PV: Data curation, Formal analysis, Validation, Writing – original draft. BK: Data curation, Formal analysis, Writing – review & editing. AH: Data curation, Formal analysis, Validation, Writing – review & editing. EM: Data curation, Formal analysis, Validation, Writing – review & editing. SS: Data curation, Writing – review & editing. MT: Conceptualization, Resources, Writing – review & editing. JC: Conceptualization, Resources, Writing – review & editing. PV: Resources, Supervision, Writing – review & editing. CdL: Conceptualization, Resources, Writing – review & editing. TC: Resources, Supervision, Writing – review & editing. LJ: Conceptualization, Funding acquisition, Supervision, Writing – review & editing. MN: Conceptualization, Funding acquisition, Supervision, Writing – review & editing.
