# Supplementary material for: Potent induction of trained immunity by Saccharomyces cerevisiae β-glucans
Source: Front Immunol. 2024 Feb 13;15:1323333. doi: 10.3389/fimmu.2024.1323333 (PMC10896952; doi:10.3389/fimmu.2024.1323333)
Supplement: Supplementary file 1 [file DataSheet_1.docx]

Supplementary Material

# Supplementary Figures and Tables

## Supplementary Figures

**
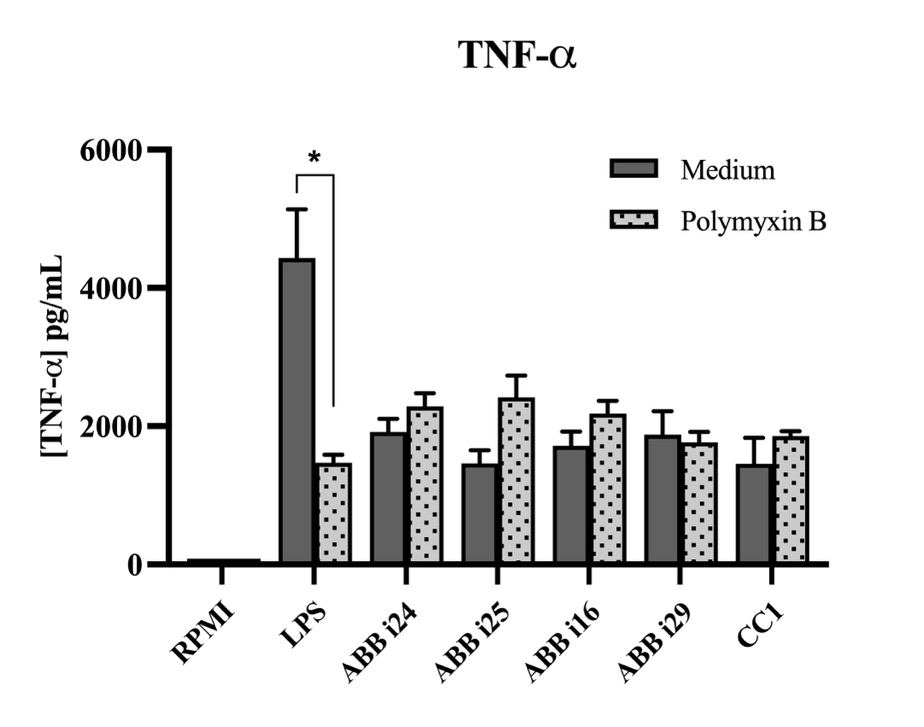
**

**Supplementary Figure 1. 24h monocyte stimulation by 5 fractions of β-glucans from *S.* *cerevisiae* in the presence of polymyxin B.** To eliminate the possibility of lipopolysaccharide (LPS) contamination, monocytes treated with ABB i24, ABB i25, ABB i16, ABB i29, and CC1 were pre-incubated with 2 ug/mL polymyxin B for 1h before monocyte stimulation for 24h. TNF production was measured in supernatants by ELISA (n=3). Data in bar plots are represented as mean ± SEM. *p < 0.05; statistical analysis was performed by Mann-Whitney U test.


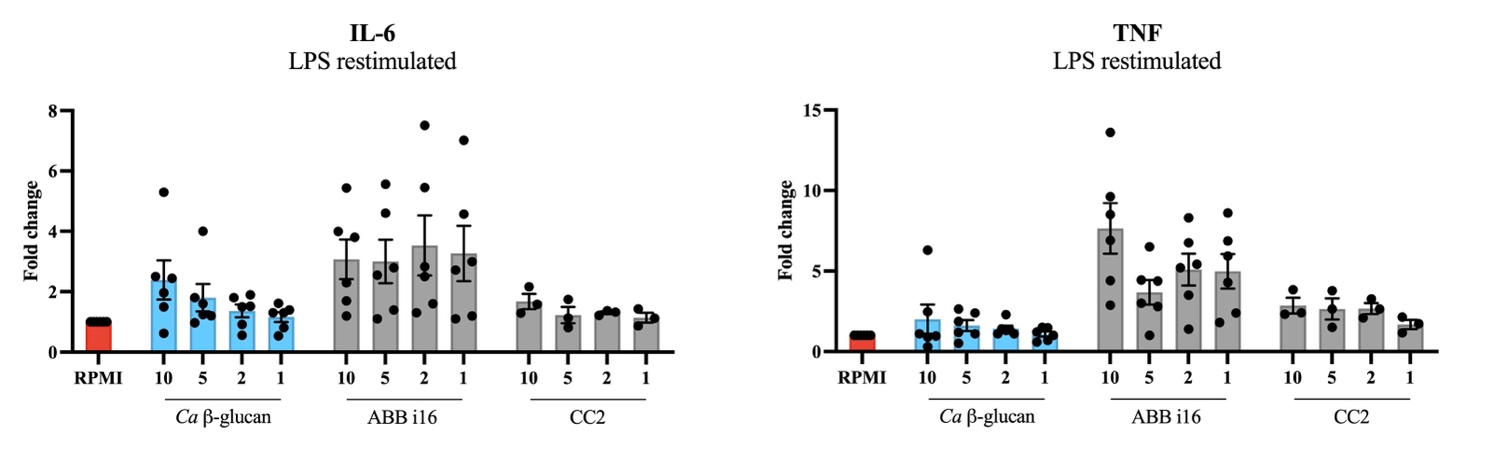


**Supplementary Figure 2. Comparison of the induction of trained immunity by ABB i16 and Wellmune CC2 beta-glucan.** Monocytes were trained with 1, 2, 5, and 10 µg/mL of *Ca* β-glucan, ABB i16, or CC2 β-glucans for 24 hours. On day 6 after resting, macrophages were re-stimulated with 10 ng/mL of LPS and incubated for 24 hours. IL-6 (**A**) and TNF (**B**) production was measured in supernatants by ELISA, n=3. Data in bar plots are represented as mean ± SEM.

**Supplementary Figure 3. Pre-treatment with ABB i16 decreases MB49 bladder cancer growth in mice.** C57BL/6 mice were i.p. injected with 1mg of ABB i16 or received a control injection (Con) 7 days before s.c. inoculation of MB49 bladder cancer cells. On day 16, tumors were extracted and their weight was measured (data presented in mg tumor weight). ** p< 0.01.
